# ﻿Two new arthroconidial yeast species from bark and pit mud in China

**DOI:** 10.3897/mycokeys.113.141799

**Published:** 2025-01-28

**Authors:** Hai-Yan Zhu, Yu-Hua Wei, Liang-Chen Guo, Zhang Wen, Shuang Hu, Di-Qiang Wang, Xiao-Long You, En-Di Fan, Shang-Jie Yao, Feng-Yan Bai, Pei-Jie Han

**Affiliations:** 1 State Key Laboratory of Mycology, Institute of Microbiology, Chinese Academy of Sciences, Beijing 100101, China Institute of Microbiology, Chinese Academy of Sciences Beijing China; 2 College of Life Sciences, University of Chinese Academy of Sciences, Beijing 100049, China University of Chinese Academy of Sciences Beijing China; 3 GuiZhou XiJiu Co., Ltd, Guizhou 564622, China GuiZhou XiJiu Co., Ltd Guizhou China

**Keywords:** Arthroconidial yeast, *Geotrichum*, *Magnusiomyces*, two new species

## Abstract

A study on yeast species from the genera *Geotrichum* and *Magnusiomyces* in southwest and central China was conducted based on morphological and molecular phylogenetic analyses using the ITS region and the D1/D2 domain of the LSU rRNA gene. The research identified two new yeast species: *Geotrichumhubeiense* and *Magnusiomycespitmudophilus*. The study contributed to understanding arthroconidial yeast diversity in fermentation and natural environments and paved the way for future taxonomic and ecological studies. Descriptions, illustrations, and phylogenetic analysis results of the two new taxa are provided.

## ﻿Introduction

The ascomycetous yeasts, or fungi that exhibit yeast-like characteristics and form arthroconidia, are categorized under the genera *Geotrichum* and *Magnusiomyces* (de Hoog and Smith 2004, 2011a, 2011b, 2011c, 2011d, 2011e; Zhu et al. 2024b). These fungi are ubiquitous and are frequently found in association with dairy products (Marcellino et al. 2001; Banjara et al. 2015), Chinese Baijiu production (Zhu et al. 2024b), marine environments (Zhu et al. 2024b), cosmetics (Kataoka et al. 2013; Takei et al. 2015), human infection (Ersoz et al. 2004; Özkaya-Parlakay et al. 2012; Fasciana et al. 2017; Shah and Mauger 2017; Tanuskova et al. 2017; Keene et al. 2019; Erman et al. 2020; Flateau et al. 2021; Tshisevhe et al. 2021; Noster et al. 2022), and plant rot (Vadkertiová et al. 2012; Gao et al. 2020; Wang et al. 2022), as well as in the emerging new energy industry (Kurylenko et al. 2020).

Early molecular phylogenetic studies utilizing sequence analyses of the LSU (Kurtzman and Robnett 1995), SSU (Ueda-Nishimura and Mikata 2000), and ITS rDNA sequences (de Hoog and Smith 2004) classified two distinct groups of arthroconidial yeasts, including the sexual genera *Dipodascus*, *Galactomyces*, and *Magnusiomyces*, and the asexual genera *Geotrichum* and *Saprochaete*. de Hoog and Smith (2004) further confirmed these groups through phylogenetic analyses of the ITS region and DNA/DNA reassociation data, validating the existence of Ribosomal Groups 1 and 2 among arthroconidial yeasts or yeast fungi in Hemiascomycetes. According to the nomenclature code adopting the “one fungus, one name” principle (McNeill et al. 2012), Zhu et al. (2024b) revised these two monophyletic groups as *Geotrichum* and *Magnusiomyces* based on the sequences of the internal transcribed spacer (ITS) region and the D1/D2 domain of the large subunit of the rDNA gene and recognised 28 species in the genus *Geotrichum* and 17 species in the genus *Magnusiomyces*. Subsequently, *Geotrichumpandrosioniae*, isolated from the dung of *Vombatusursinus*, and *Dipodascushypatia* isolated from soil under *Zingiberzerumbet*, were described by Tan and Shivas (2023) from Australia. *Geotrichumenheduannae* from the scat of *Casuariuscasuarius* was also described by Tan and Shivas (2024) from Australia.

China, known for its rich biodiversity, is one of the most biodiverse countries in the world. (The Biodiversity Committee of Chinese Academy of Sciences, 2024). However, compared to the extensive studies on plants and animals, fungal diversity, particularly yeasts, has received significantly less attention. The ‘Catalogue of Life China: 2024 Annual Checklist’ documents a comprehensive count of 69,407 species within the Animalia kingdom, 39,897 species within the Plantae kingdom, and 26,591 species within the Fungi kingdom. Boekhout et al. (2022) reported that at least 782 yeast species were known in China by 2020, and 125 new yeast species were discovered in the subsequent four years (Gao et al. 2021; Liu et al. 2021, 2023, 2024c, 2024a, 2024b; Shi et al. 2021; Chai et al. 2022b, 2022c, 2022a, 2023, 2024; Chu et al. 2022; Hu et al. 2022; Wei et al. 2022, 2024a, 2024b; Qiao et al. 2023, 2024; Yu et al. 2023; Zhu et al. 2023a, 2023b, 2024b, 2024a; Cai et al. 2024; Guo et al. 2024a, 2024b; Jiang et al. 2024; Lu et al. 2024; Xi et al. 2024). Furthermore, based on DNA metabarcoding analyses, they estimated that the natural world likely hosts over 20,000 yeast species. This suggests that many yeast species remain to be discovered, highlighting the vast potential for future exploration in this field.

During our surveys of yeast diversity in samples from traditional fermentation and terrestrial natural environments, five isolates representing two arthroconidial yeast species in *Geotrichum* and *Magnusiomyces* were identified based on morphology and molecular phylogenetic analyses, which increased the species diversity of arthroconidial yeast species in China.

## ﻿Materials and methods

### ﻿Sample collection and yeast isolation

Twenty traditional fermentation environment samples and nine terrestrial natural environment samples were collected, and important collection information was noted (Rathnayaka et al. 2024) from southwest China, namely, Guizhou Province, in May 2023, and central China, namely, Hubei Province, in July 2023, respectively. All samples were placed into sterile sampling bags, transferred to the laboratory at 25 ± 2 °C, and subjected to yeast isolation. The pit mud samples, containing rich microbial communities used in the traditional Chinese Baijiu brewing process, were processed by suspending 60 grams in a 250 mL conical flask containing 180 mL of sterile water and shaking at 200 rpm for 30 minutes at 30 °C. Subsequently, each suspension was diluted to 1 × 10^-1^, and 100 μL of stock and dilution was plated on yeast extract peptone dextrose agar (YPD, w/v, 2% glucose, 1% yeast extract, 2% peptone, and 2% agar) plates supplemented with 200 µg/mL chloramphenicol. Plates were incubated at 30 °C for 3–5 days. The moss-covered bark samples from unknown plants were enriched and isolated using the method described by Zhu et al. (2023c). Yeast and yeast-like colonies on the plates were picked, purified, and preserved in 25% glycerol at –80 °C.

### ﻿Phenotypic characterization

Morphological characteristics and physiological and biochemical properties were examined according to standard methods described by Kurtzman et al. (2011). The assimilation of carbon and nitrogen compounds was conducted in liquid media. The potential sexual cycles of strains representing new species were investigated using corn meal agar (CMA, w/v, 2.5% corn starch and 2% agar), potato dextrose agar (PDA, w/v, 20% potato infusion, 2% glucose, and 2% agar), yeast extract–malt extract agar (YM, w/v, 1% glucose, 0.3% yeast extract, 0.3% malt extract, 0.5% peptone, and 2% agar), V8 agar (w/v, 10% V8 juice and 2% agar), and yeast carbon base agar (YCB, w/v, 1.17% yeast carbon base and 2% agar). A loopful of cells of each test strain was inoculated separately or mixed on agar plates, incubated at 25 °C for up to two months, and examined periodically.

### ﻿DNA extraction, sequencing, and phylogenetic analyses

A sesame seed-sized quantity of fresh yeast cells was transferred to 70 µL of sterile 0.1 M sodium hydroxide solution, where they were subjected to lysis at 98 °C for 15 minutes to extract yeast genomic DNA. The ITS region and D1/D2 domain of the LSU rRNA gene were amplified using primers ITS1 and ITS4 (White et al. 1990) and NL1 and NL4 (O’Donnell 1993), respectively, and were also sequenced using the methods described by Bai et al. (2002). Sequence alignments were conducted with MAFFT v.7 (Katoh and Standley 2013), with ambiguous positions excluded using GBLOCKS v.0.91b (Castresana 2000). Phylogenetic analysis based on single ITS or D1/D2 sequences was executed utilizing the Neighbor-Joining model in MEGA v.7 with evolutionary distances derived from Kimura’s two-parameter model (Kimura 1980; Kumar et al. 2016; Lachance 2022). Bootstrap analyses were performed on 1,000 random re-sampling (Felsenstein 1985). Maximum parsimony (MP), Bayesian Inference (BI), and maximum likelihood (ML) analyses were conducted on the combined ITS and D1/D2 sequences using PAUP v.4.0b10 (Swofford 2003), MRBAYES v.3.2 with 1,000,000 generations (Ronquist et al. 2012), and RAXML-HPC 7.2.8 with 1,000 bootstrap replicates (Stamatakis 2006), respectively. The optimal nucleotide substitution model was estimated using MODELTEST v.3.04 (Posada and Crandall 1998), with the GTR + I + G model selected for the ML and BI analyses.

## ﻿Results

### ﻿Phylogenetic analyses

A total of five yeast strains from pit mud and bark samples were preliminarily identified as two novel arthroconidial yeast species in the genera *Geotrichum* and *Magnusiomyces* using the BLAST search tool based on the ITS and D1/D2 sequences against the NCBI GenBank database. Besides the newly generated sequences, additional related sequences (Suppl. material 3) were also downloaded from GenBank for the phylogenetic analyses.

**Figure 1.**
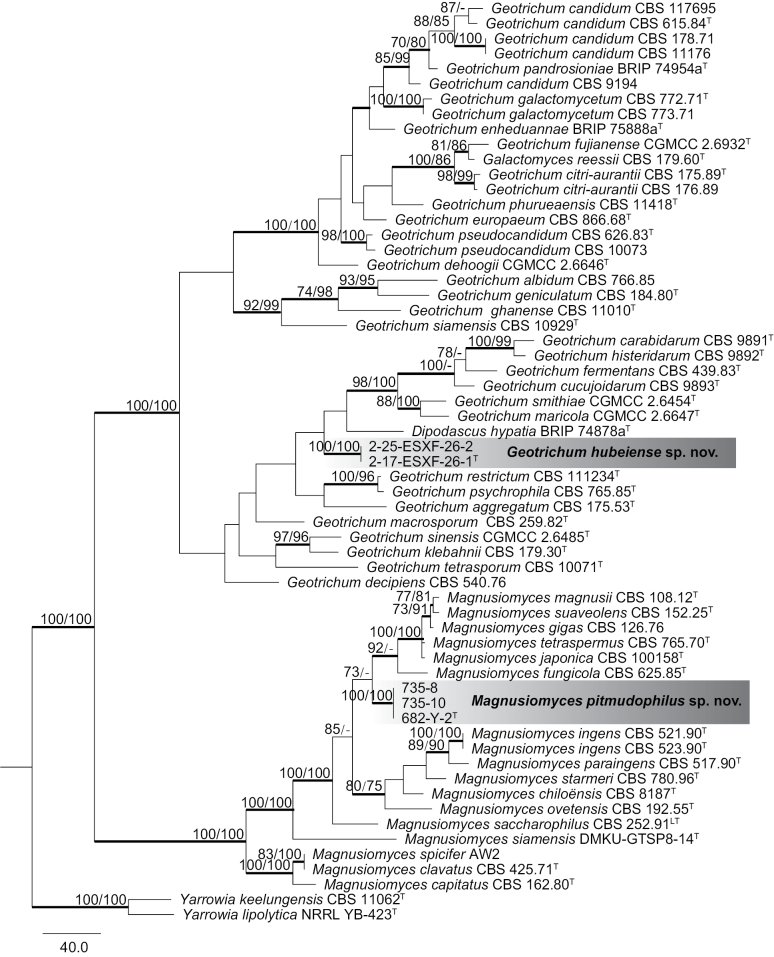
Phylogeny of arthroconidial yeast species based on maximum parsimony (MP) analysis of the combined ITS and D1/D2 sequences. The two *Yarrowia* species were used as the outgroup. The MP/maximum likelihood (ML) bootstrap support values equal to or above 70% are shown. Bold lines represent posterior probabilities equal to or above 0.95 from the Bayesian Inference (BI) test. Type strains are marked with the superscript “T”. Lectotype strain is marked with the superscript “LT”.

Strains 2-25-ESXF-26-2 and 2-17-ESXF-26-1^T^ from the same bark samples in Hubei province possessed identical ITS sequences and similar D1/D2 sequences with one nucleotide substitution, indicating that they were conspecific. The phylogenetic analyses based on the D1/D2 and ITS sequences confirmed the affinity of the 2-17-ESXF-26-1^T^ group to the genus *Geotrichum* with high bootstrap support (Fig. 1, Suppl. materials 1, 2). However, the phylogenetic position of this group within the genus was not consistently resolved when the combined ITS and D1/D2 sequences and single ITS or D1/D2 sequences were used (Fig. 1, Suppl. materials 1, 2). The 2-17-ESXF-26-1^T^ group was closely related to *Dipodascushypatia* in the *Geotrichum* clade in the tree based on the D1/D2 sequences (Suppl. material 1) but exhibited a close relationship with *G.carabidarum*, *G.histeridarum*, and *Dipodascushypatia* in the ITS tree (Suppl. material 2). The bootstrap support for the phylogenetic relationships of the 2-17-ESXF-26-1^T^ group with the described species of the genus *Geotrichum* was lower than 70% in the ITS tree (Suppl. material 2) but 100% in the D1/D2 tree (Suppl. material 1). The results of pairwise comparisons indicated that strain 2-17-ESXF-26-1^T^ exhibited 84 (15.2%, 67 substitutions and 17 gaps, total length: 552) nucleotide differences and 37 (14.0%, 23 substitutions and 14 gaps, total length: 265) nucleotide differences from its closely related species *Dipodascushypatia* in the D1/D2 domain and ITS region, respectively. Strain 2-17-ESXF-26-1^T^ possessed 86 (16.1%, 58 substitutions and 28 gaps, total length: 533) nucleotide differences and 38 (15.8%, 22 substitutions and 16 gaps, total length: 241) nucleotide differences from its closely related species *Geotrichumhisteridarum* in the D1/D2 domain and ITS region, respectively. Strain 2-17-ESXF-26-1^T^ possessed 95 (17.2%, 58 substitutions and 37 gaps, total length: 553) nucleotide differences and 38 (14.4%, 24 substitutions and 14 gaps, total length: 264) nucleotide differences from its closely related species *Geotrichumcarabidarum* in the D1/D2 domain and ITS region, respectively. The results indicated that the 2-17-ESXF-26-1^T^ group represents a novel species of the genus *Geotrichum*, for which the name *G.hubeiense* is proposed.

Strains 735-8, 735-10, and 682-Y-2^T^, isolated from two pit mud samples collected from different brewing workshops of a well-known Chinese Baijiu enterprise in Zunyi City, Guizhou Province, possessed identical ITS and D1/D2 sequences, thus suggesting their conspecificity. Strains 735-8 and 735-10 were isolated from one workshop, while strain 682-Y-2^T^ was isolated from another. In the combined D1/D2 and ITS tree and the single ITS tree, the group 682-Y-2 and other six species, namely, *Magnusiomycesfungicola*, *M.gigas*, *M.japonica*, *M.magnusii*, *M.suaveolens*, and *M.tetraspermus*, grouped in a branch in the genus *Magnusiomyces* (Fig. 1, Suppl. material 2). However, in the D1/D2 tree, the aforementioned branch newly added the species *M.saccharophilus* besides the six species (Suppl. material 1). The phylogenetic positions of the species *M.saccharophilus* were not resolved when the combined ITS and D1/D2 sequences and single ITS or D1/D2 sequences were used (Fig. 1, Suppl. material 1, 2). The group 682-Y-2^T^ differed from the type strains of the described species in the branch, including the seven species, by 8 to 18 (2.0–4.5%) nucleotide differences and 14 to 20 (5.5–7.6%) nucleotide differences in the D1/D2 domain and ITS region, respectively (Table 1). These results suggested that the three strains represent a novel species in the *Magnusiomyces* genus, for which *M.pitmudophilus* is proposed.

**Table 1.** Sequence differences between *M.pitmudophilus* sp. nov. and the type strains of the closely related species.

Species	D1/D2 domain		ITS region	
	Difference (%)	Substitutions/gaps/ total length	Difference (%)	Substitutions/gaps/ total length
*M.fungicola*	2.8	10/1/400	7.5	13/7/265
*M.gigas*	2.5	10/0/399	5.5	9/5/254
*M.japonica*	2.2	8/1/400	5.5	10/4/253
*M.magnusii*	2.0	8/0/399	5.5	9/5/254
*M.saccharophilus*	4.5	18/0/399	7.6	14/5/249
*M.suaveolens*	2.5	10/0/399	5.9	8/7/256
*M.tetraspermus*	3.5	14/0/399	6.3	11/5/254

### ﻿Taxonomy

#### ﻿ Geotrichum hubeiense

Taxon classificationFungiSaccharomycetalesDipodascaceae

L.C. Guo, H.Y. Zhu, P.J. Han & F.Y. Bai sp. nov.

829AD567-F840-50C8-8D3B-C136B38D5B01

Fungal Names: FN 572209

Fig. 2

##### Etymology.

The species is named after the location “Hubei Province, China,” where the type strain of the species was collected.

##### Holotype.

China • Hubei Province, Enshi City, Xianfeng County, from a bark sample, on July 7, 2023, L.C. Guo, (holotype CGMCC 2.7499^T^, permanently preserved in a metabolically inactive state, ex-holotype JCM 36896 = 2-17-ESXF-26-1).

##### Description.

**Figure 2.**
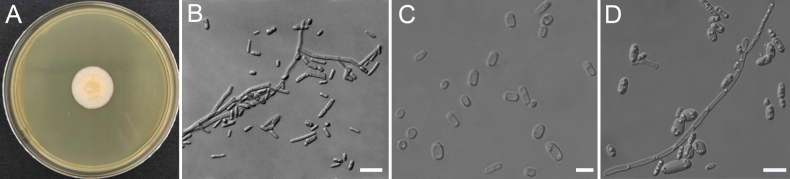
Morphology of *G.hubeiense* sp. nov. (Strain CGMCC 2.7499^T^) **A** colonies on yeast extract peptone dextrose (YPD) after 10 days **B** cylindrical arthroconidia on yeast extract–malt extract (YM) **C** cylindrical arthroconidia on V8 agar **D** blastoconidia on potato dextrose agar (PDA). Scale bars: 10 μm (**B−D**).

***Culture characteristics***: After 10 days on YPD agar at 30 °C, colonies are 22 mm in diameter, flesh colour, flat, dry, with finely hairy and regular margins (Fig. 2A). Hyphae soon disarticulate into cubic arthroconidia measuring 2.1–5.2 × 3.8–9.8 μm (Fig. 2B−C). Hyphae and arthroconidia produce oblong blastoconidia measuring 2.9–6.8 × 5.2–17.2 μm, which were observed on PDA and YCB agar after one month at 25 °C (Fig. 2D). Sexual structures were not observed on YCB, PDA, V8, YM, and CMA agar. ***Physiological and biochemical characteristics***: Glucose is not fermented. Glucose, D-galactose, L-sorbose, D-xylose, ethanol, glycerol, D-mannitol, D-glucitol, DL-lactic acid, succinic acid (weak), and citric acid (weak) are assimilated as sole carbon sources. Trehalose, ribitol, sucrose, D-maltose, cellobiose (slow), lactose, melibiose, raffinose, melezitose, inulin, starch soluble, L-arabinose, D-arabinose, D-ribose, L-rhamnose, D-glucosamine, methanol, erythritol, galactitol, α-methyl-D-glucoside, salicin, D-glucuronic acid, inositol, hexadecane, N-acetyl-D-glucosamine, and xylitol are not assimilated as sole carbon sources. Ethylamine, cadaverine, ammonium sulfate, L-lysine, potassium nitrate (late), and sodium nitrite (weak) are assimilated as sole nitrogen sources. Urease activity is negative. Diazonium Blue B reaction is negative. Extracellular starch compounds are not produced. No growth occurs in 10% (w/v) sodium chloride plus 5% (w/v) glucose medium. Growth occurs on 60% (w/v) glucose-yeast extract agar. Growth in vitamin-free medium is positive. Growth occurs on YPD agar at 35 °C, but not at 37 °C.

##### Materials examined.

China • Hubei Province, Enshi City, Xianfeng County, from a bark sample, in July 2023, L.C. Guo, living culture 2-25-ESXF-26-2 = CGMCC 2.7418 = JCM 36897.

##### Notes.

Physiologically, *G.hubeiense* sp. nov. differs from its closely related species, *G.carabidarum* and *G.histeridarum*, in its ability to assimilate D-galactose and grow on 60% (w/v) glucose-yeast extract agar.

#### ﻿ Magnusiomyces pitmudophilus

Taxon classificationFungiSaccharomycetalesDipodascaceae

Y.H. Wei, H.Y. Zhu, P.J. Han & F.Y. Bai sp. nov.

AE44CF54-D55B-5F8F-81E9-56B7B19C9CC0

Fungal Names: FN 572210

Fig. 3

##### Etymology.

The species is named after the isolation source, pit mud.

##### Holotype.

China • Guizhou Province, Zunyi City, Xishui County, from a pit mud sample, on May 8, 2023, X.L. You and E.D. Fan, (holotype CGMCC 2.7496^T^ permanently preserved in a metabolically inactive state, ex-holotype JCM 36982 = 682-Y-2).

##### Description.

**Figure 3.**
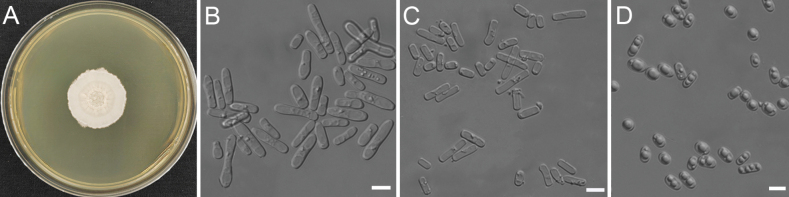
Morphology of *M.pitmudophilus* sp. nov. (Strain CGMCC 2.7496^T^) **A** colonies on yeast extract peptone dextrose (YPD) after 10 days **B** cylindrical arthroconidia on yeast extract–malt extract (YM) **C** cylindrical arthroconidia on V8 agar **D** blastoconidia on potato dextrose agar (PDA). Scale bars: 10 μm (**B−D**).

***Culture characteristics***: After 10 days on YPD agar at 30 °C, colonies are 28 mm in diameter, white, dry, and powdery, with finely hairy and irregular margins (Fig. 3A). Hyphae soon disarticulate into cubic arthroconidia measuring 3.3–7.7 × 8.1–29.0 μm (Fig. 3B−C). Hyphae and arthroconidia produce oblong blastoconidia measuring 5.3–8.3 × 7.2–15.2 μm on PDA agar after one month at 25 °C (Fig. 3D). Sexual structures were not observed on YCB, PDA, V8, YM, and CMA agar. ***Physiological and biochemical characteristics***: Glucose is not fermented. Glucose, D-galactose, L-sorbose, ethanol, glycerol, D-glucitol, D-mannitol, and DL-lactic acid (weak) are assimilated as sole carbon sources. Trehalose, ribitol, succinic acid, citric acid, sucrose, D-maltose, cellobiose, lactose, melibiose, raffinose, melezitose, inulin, starch soluble, D-xylose, L-arabinose, D-arabinose, D-ribose, L-rhamnose, D-glucosamine, methanol, erythritol, galactitol, α-methyl-D-glucoside, salicin, D-glucuronic acid, inositol, hexadecane, N-acetyl-D-glucosamine, and xylitol are not assimilated as sole carbon sources. Ethylamine, cadaverine, ammonium sulfate, L-lysine, potassium nitrate (weak), and sodium nitrite (weak) are assimilated as sole nitrogen sources. Urease activity is positive. Diazonium Blue B reaction is negative. Extracellular starch compounds are not produced. No growth occurs in 10% (w/v) sodium chloride plus 5% (w/v) glucose medium. Growth occurs on 50% (w/v) glucose-yeast extract agar. No growth occurs on 60% (w/v) glucose-yeast extract agar. Growth in vitamin-free medium is positive. Growth occurs on YPD agar at 35 °C, but not at 37 °C.

##### Materials examined.

China • Guizhou Province, Zunyi City, Xishui County, a pit mud sample, on May 8, 2023, X.L. You and E.D. Fan, living culture 735-8 = CGMCC 2.7774 = JCM 36984; • *ibid*. living culture 735-10 = CGMCC 2.7775.

##### Notes.

Physiologically, *M.pitmudophilus* sp. nov. differs from its closely related species *M.fungicola* and *M.japonica* in its inability to assimilate succinic acid, from *M.suaveolens* and *M.saccharophilus* in its inability to ferment glucose, from *M.gigas* in its inability to ferment both glucose and galactose, from *M.magnusii* in its inability to ferment glucose, galactose, and sucrose, and from *M.tetraspermus* in its inability to ferment glucose and grow at 37 °C (Table 2). The three strains representing *M.pitmudophilus* sp. nov. are from pit mud collected in China, suggesting the unique niche of the new species.

**Table 2.** Salient phenotypical characteristics distinguishing *M.pitmudophilus* sp. nov. from the closely related species. NA: not available.

Species	Fermentation			Assimilation	Growth		
	Glucose	Galactose	Sucrose	Succinate	35 °C	37 °C	40 °C
*M.pitmudophilus* sp. nov.	−	−	−	−	+	−	−
*M.fungicola*	−	−	−	+	+	w	−
*M.gigas*	+	+	−	+	+	−	−
*M.japonica*	NA	NA	NA	+	+	−	−
*M.magnusii*	+	+/w	+	+	−	−	−
*M.saccharophilus*	+	v	−	+/w	+	−	−
*M.suaveolens*	+	v	−	+/w	+	−	−
*M.tetraspermus*	+	−	−	+	+	+	+

## ﻿Discussion

In this study, two novel species, *Geotrichumhubeiense* and *Magnusiomycespitmudophilus*, were recognized based on ITS and D1/D2 sequence analyses. Currently, 30 *Geotrichum* species and 17 *Magnusiomyces* species have been accepted (Fig. 1, Suppl. material 3).

Arthroconidial yeast species are found globally in diverse environments, including wild and fermentation (Suppl. material 3). In the present study, we isolated the first novel species, *G.hubeiense*, from the bark in the Hubei Province of China. Other species, such as *Geotrichumalbidum*, *G.klebahnii*, *G.macrosporum*, *G.psychrophila*, *Magnusiomycesjaponicus*, *M.quercus*, and *M.magnusii*, were all sourced from slime. *Geotrichumgeniculatum* was isolated from guava, and *Geotrichumrestrictum* from spruce (Suppl. material 3). They are all from plant-associated sources. The marine environment also hosts a variety of arthroconidial yeast species. Zhu et al. (2024b) described five *Geotrichum* species from marine habitats. *Geotrichumtetrasporum* was found in deep-sea sediments (de Hoog and Smith 2011b). Additionally, the guts of insects and soil are common sources for these species (Suppl. material 3). The diversity of these sources highlights the broad ecological adaptability of arthroconidial yeast species in various natural environments.

Meanwhile, we isolated the second novel species, *M.pitmudophilus*, and two known species, *Geotrichumcandidum*, aligning with findings reported by Zhu et al. (2024b) and *Geotrichumeuropaeum*, both of which were extracted from the pit mud (data not shown). Pit mud offers a good medium for microbial growth and plays a significant role in the process of Chinese Baijiu brewing (Pan et al. 2023). In addition to the three species mentioned above, *Magnusiomycesingens* and *Magnusiomycesparaingens* were also isolated from the wine cellar, an environment intrinsically linked to wine production (de Hoog and Smith 2011e). Beyond the fermentation milieu specific to wine, other distinct fermentation settings have been explored, such as the use of *Geotrichumghanense* in the fermentation of cocoa (Nielsen et al. 2010) and *Geotrichumcandidum* in dairy fermentation processes (Sulo et al. 2009; Groenewald et al. 2012). These results suggest that several arthroconidial yeast species occupy important ecological niches in the fermented environment and play crucial roles.

## Supplementary Material

XML Treatment for Geotrichum hubeiense

XML Treatment for Magnusiomyces pitmudophilus

## ﻿Additional information

### Author ORCIDs

Hai-Yan Zhu https://orcid.org/0000-0001-5547-4922

Yu-Hua Wei https://orcid.org/0000-0001-5789-5683

Liang-Chen Guo https://orcid.org/0009-0004-9404-4449

Shuang Hu https://orcid.org/0000-0001-7285-7626

Feng-Yan Bai https://orcid.org/0000-0001-9823-6430

Pei-Jie Han https://orcid.org/0000-0001-8342-739X

### Data availability

All of the data that support the findings of this study are available in the main text or Supplementary Information.

## Supplementary materials

Supplementary material 1 Phylogeny of the described arthroconidial yeast species based on neighbor-joining (NJ) analysis of the D1/D2 sequences Data type pdf Explanation note The two *Yarrowia* species were used as the outgroup. The NJ bootstrap support values equal to or above 70% are shown. Type strains are marked with the superscript “T”. Lectotype strain is marked with the superscript “LT”. https://binary.pensoft.net/file/1236703This dataset is made available under the Open Database License (http://opendatacommons.org/licenses/odbl/1.0/). The Open Database License (ODbL) is a license agreement intended to allow users to freely share, modify, and use this Dataset while maintaining this same freedom for others, provided that the original source and author(s) are credited.Hai-Yan Zhu, Yu-Hua Wei, Liang-Chen Guo, Zhang Wen, Shuang Hu, Di-Qiang Wang, Xiao-Long You, En-Di Fan, Shang-Jie Yao, Feng-Yan Bai, Pei-Jie Han

Supplementary material 2 Phylogeny of the arthroconidial yeast species based on neighbor-joining (NJ) analysis of the ITS sequences Data type pdf Explanation note The two *Yarrowia* species were used as the outgroup. The NJ bootstrap support values equal to or above 70% are shown. Type strains are marked with the superscript “T”. Lectotype strain is marked with the superscript “LT”. https://binary.pensoft.net/file/1236704This dataset is made available under the Open Database License (http://opendatacommons.org/licenses/odbl/1.0/). The Open Database License (ODbL) is a license agreement intended to allow users to freely share, modify, and use this Dataset while maintaining this same freedom for others, provided that the original source and author(s) are credited.Hai-Yan Zhu, Yu-Hua Wei, Liang-Chen Guo, Zhang Wen, Shuang Hu, Di-Qiang Wang, Xiao-Long You, En-Di Fan, Shang-Jie Yao, Feng-Yan Bai, Pei-Jie Han

Supplementary material 3 The names, strain numbers, sources, locations, and corresponding GenBank accession numbers of the yeast taxa employed in this study Data type xls Explanation note All the new isolates used in this study are indicated in bold. Superscript T: Type strain. Superscript LT: Lectotype strain. Superscript IT: Isotype strain. *: Sequences obtained from the genome. https://binary.pensoft.net/file/1236705This dataset is made available under the Open Database License (http://opendatacommons.org/licenses/odbl/1.0/). The Open Database License (ODbL) is a license agreement intended to allow users to freely share, modify, and use this Dataset while maintaining this same freedom for others, provided that the original source and author(s) are credited.Hai-Yan Zhu, Yu-Hua Wei, Liang-Chen Guo, Zhang Wen, Shuang Hu, Di-Qiang Wang, Xiao-Long You, En-Di Fan, Shang-Jie Yao, Feng-Yan Bai, Pei-Jie Han

## References

[B1] Bai FY Zhao JH Takashima M Jia JH Boekhout T Nakase T (2002) Reclassification of the *Sporobolomycesroseus* and *Sporidioboluspararoseus* complexes, with the description of *Sporobolomycesphaffii* sp. nov. International Journal of Systematic and Evolutionary Microbiology 52: 2309–2314. 10.1099/00207713-52-6-2309 12508902

[B2] Banjara N Suhr MJ Hallen-Adams HE (2015) Diversity of yeast and mold species from a variety of cheese types. Current Microbiology 70: 792–800. 10.1007/s00284-015-0790-1 25694357

[B3] Boekhout T Amend AS El Baidouri F Gabaldón T Geml J Mittelbach M Robert V Tan CS Turchetti B Vu D Wang QM Yurkov A (2022) Trends in yeast diversity discovery. Fungal Diversity 114: 491–537. 10.1007/s13225-021-00494-6

[B4] Cai DY Liu S Hui FL (2024) *Kondoatianchiensis* f.a., sp. nov., an anamorphic yeast species isolated from plant leaves. International Journal of Systematic and Evolutionary Microbiology 74: 1–6. 10.1099/ijsem.0.006487 39661431

[B5] Castresana J (2000) Selection of conserved blocks from multiple alignments for their use in phylogenetic analysis. Molecular Biology and Evolution 17: 540–552. 10.1093/oxfordjournals.molbev.a026334 10742046

[B6] Chai CY Gao WL Yan ZL Hui FL (2022a) Four new species of Trichomonascaceae (Saccharomycetales, Saccharomycetes) from Central China. MycoKeys 90: 1–18. 10.3897/mycokeys.90.83829 36760421 PMC9849089

[B7] Chai CY Li Y Yan ZL Hui FL (2022b) Phylogenetic and genomic analyses of two new species of *Clavispora* (Metschnikowiaceae, Saccharomycetales) from Central China. Frontiers in Microbiology 13: 1–11. 10.3389/fmicb.2022.1019599 PMC9608443 36312955

[B8] Chai CY Gao WL Li Y Yan ZL Hui FL (2022c) *Kodamaeahongheensis* f.a., sp. nov., *Kodamaeaovata* f.a., sp. nov. and *Kodamaeayamadae* f.a., sp. nov., three new yeast species of *Kodamaea* (Saccharomycetales, Debaryomycetacae) from China. MycoKeys 89: 121–137. 10.3897/mycokeys.89.81119 36760829 PMC9849074

[B9] Chai CY Lei T Chu XY Hui FL (2023) Multi-gene phylogeny and taxonomy of the genus *Bannoa* with the addition of three new species from central China. Frontiers in Microbiology 14: 1–10. 10.3389/fmicb.2023.1143156 PMC10043259 36998405

[B10] Chai CY Ke T Niu QH Hui FL (2024) Diversity of *Wickerhamomyces* (Wickerhamomycetaceae, Saccharomycetales) in China with the description of four new species. Frontiers in Microbiology 15: 1–13. 10.3389/fmicb.2024.1338231 PMC10881795 38389540

[B11] Chu SB Hu WT Hui FL (2022) *Torulasporajiuxiensis* sp. nov., a novel yeast species isolated from rotting wood. International Journal of Systematic and Evolutionary Microbiology 72: 005629. 10.1099/ijsem.0.005629 36748467

[B12] de Hoog GS Smith MT (2004) Ribosomal gene phylogeny and species delimitation in *Geotrichum* and its teleomorphs. Studies in Mycology 50: 489–515.

[B13] de Hoog GS Smith MT (2011a) *Galactomyces* Redhead & Malloach (1977). In: Kurtzman C Fell J Boekhout T (Eds) The yeasts, a taxonomic study.5^th^ edn. Elsevier, Amsterdam, 413–420. 10.1016/B978-0-444-52149-1.00031-8

[B14] de Hoog GS Smith MT (2011b) *Dipodascus* de Lagerheim (1892). In: Kurtzman C Fell J Boekhout T (Eds) The yeasts, a taxonomic study.5^th^ edn. Elsevier, Amsterdam, 385–395.

[B15] de Hoog GS Smith MT (2011c) *Geotrichum* link: Fries (1832). In: Kurtzman C Fell J Boekhout T (Eds) The yeasts, a taxonomic study.5^th^ edn. Elsevier, Amsterdam, 1279–1286. 10.1016/B978-0-444-52149-1.00091-4

[B16] de Hoog GS Smith MT (2011d) *Magnusiomyces* Zender (1977). In: Kurtzman C Fell J Boekhout T (Eds) The yeasts, a taxonomic study.5^th^ edn. Elsevier, Amsterdam, 565–574. 10.1016/B978-0-444-52149-1.00045-8

[B17] de Hoog GS Smith MT (2011e) *Saprochaete* Coker & Shanor ex D. T. S. Wagner & Daws (1970). In: Kurtzman C Fell J Boekhout T (Eds) The yeasts, a taxonomic study.5^th^ edn. Elsevier, Amsterdam, 1317–1327. 10.1016/B978-0-444-52149-1.00097-5

[B18] Erman B Fırtına S Aksoy BA Aydogdu S Genç GE Doğan Ö Bozkurt C Fışgın T Çipe FE (2020) Invasive *Saprochaetecapitata* infection in a patient with autosomal recessive CARD9 deficiency and a review of the literature. Journal of Clinical Immunology 40: 466–474. 10.1007/s10875-020-00759-w 32020378

[B19] Ersoz G Otag F Erturan Z Aslan G Kaya A Emekdas G Sugita T (2004) An Outbreak of *Dipodascuscapitatus* infection in the ICU: three case reports and review of the literature. Japanese Journal of Infectious Diseases 57: 248–252. 10.7883/yoken.JJID.2004.248 15623948

[B20] Fasciana T Giuffrè M Calà C Schierz IAM Aquilina G Pinello G Capra G Lipari D Corsello G Giammanco A (2017) Genotyping and antifungal susceptibility of Dipodascuscapitatus isolated in a neonatal intensive care unit of a sicilian hospital. Advances in Experimental Medicine and Biology 973: 81–88. 10.1007/5584_2016_195 28213808

[B21] Felsenstein J (1985) Confidence limits on phylogenies: an approach using the bootstrap. Evolution 39: 783–791. 10.2307/2408678 28561359

[B22] Flateau C Aït-Ammar N Angebault C Salomon L Matignon M Lepeule R Melica G Grimbert P Lelièvre JD Gallien S Botterel F (2021) Risk factors for intra-abdominal fungal infection after simultaneous pancreas-kidney transplantation: A single-center retrospective experience. Transplant Infectious Disease 23: 1–10. 10.1111/tid.13486 33047447

[B23] Gao H Yin X Jiang X Shi H Yang Y Wang C Dai X Chen Y Wu X (2020) Diversity and spoilage potential of microbial communities associated with grape sour rot in eastern coastal areas of China. PeerJ 2020: 1–17. 10.7717/peerj.9376 PMC7315622 32607286

[B24] Gao WL Li Y Chai CY Yan ZL Hui FL (2021) New species of *Yamadazyma* from rotting wood in China. MycoKeys 83: 69. 10.3897/mycokeys.83.71156 PMC8413266 34539207

[B25] Groenewald M Coutinho T Smith MT van der Walt JP (2012) Species reassignment of *Geotrichumbryndzae*, *Geotrichumphurueaensis*, *Geotrichumsilvicola* and *Geotrichumvulgare* based on phylogenetic analyses and mating compatibility. International Journal of Systematic and Evolutionary Microbiology 62: 3072–3080. 10.1099/ijs.0.038984-0 22798647

[B26] Guo QC Liu S Hui FL (2024a) *Spencermartinsiellahenanensis* fa., sp. nov., a novel yeast species isolated from rotting wood. International Journal of Systematic and Evolutionary Microbiology 74: 1–5. 10.1099/ijsem.0.006226 38190334

[B27] Guo QC Liu S Qiao YZ Hui FL (2024b) Three new species of *Teunia* (Cryptococcaceae, Tremellales) identified through phenotypic and phylogenetic analyses. MycoKeys 105: 139–153. 10.3897/mycokeys.105.120534 38783907 PMC11112159

[B28] Hu WT Chu SB Li Y Hui FL (2022) *Hyphopichiaxiaguanensis* f.a., sp. nov., an ascomycetous yeast species isolated from plant leaves. International Journal of Systematic and Evolutionary Microbiology 72(5). 10.1099/ijsem.0.005398 35617012

[B29] Jiang YL Bao WJ Liu F Wang GS Yurkov AM Ma Q Hu ZD Chen XH Zhao WN Li AH Wang QM (2024) Proposal of one new family, seven new genera and seventy new basidiomycetous yeast species mostly isolated from Tibet and Yunnan provinces, China. Studies in Mycology 109: 57–153. 10.3114/sim.2024.109.02 39717653 PMC11663428

[B30] Kataoka S Hattori K Date A Tamura H (2013) Human keratinocyte caspase-14 expression is altered in human epidermal 3D models by dexamethasone and by natural products used in cosmetics. Archives of Dermatological Research 305: 683–689. 10.1007/s00403-013-1359-0 23604963

[B31] Katoh K Standley DM (2013) MAFFT multiple sequence alignment software version 7: Improvements in performance and usability. Molecular Biology and Evolution 30: 772–780. 10.1093/molbev/mst010 23329690 PMC3603318

[B32] Keene S Sarao MS McDonald PJ Veltman J (2019) Cutaneous geotrichosis due to *Geotrichumcandidum* in a burn patient. Access Microbiology 1. 10.1099/acmi.0.000001 PMC7470357 32974489

[B33] Kimura M (1980) A simple method for estimating evolutionary rates of base substitutions through comparative studies of nucleotide sequences. Journal of Molecular Evolution 16: 111–120. 10.1007/BF01731581 7463489

[B34] Kumar S Stecher G Tamura K (2016) MEGA7: Molecular Evolutionary Genetics Analysis Version 7.0 for Bigger Datasets. Molecular Biology and Evolution 33: 1870–1874. 10.1093/molbev/msw054 27004904 PMC8210823

[B35] Kurtzman CP Robnett CJ (1995) Molecular relationships among hyphal ascomycetous yeasts and yeastlike taxa. Canadian Journal of Botany 73: 824–830. 10.1139/b95-328

[B36] Kurtzman CP Fell JW Boekhout T Robert V (2011) Methods for isolation, phenotypic characterization and maintenance of yeasts. In: Kurtzman C Fell J Boekhout T (Eds) The yeasts, a taxonomic study.5^th^ edn. Elsevier, Amsterdam, 87–110. 10.1016/B978-0-444-52149-1.00007-0

[B37] Kurylenko OO Ruchala J Dmytruk K V. Abbas CA Sibirny AA (2020) Multinuclear Yeast *Magnusiomyces* (*Dipodascus*, *Endomyces*) *magnusii* is a promising isobutanol producer. Biotechnology Journal 15: 1–8. 10.1002/biot.201900490 31990438

[B38] Lachance MA (2022) Phylogenies in yeast species descriptions: In defense of neighbor-joining. Yeast 39: 513–520. 10.1002/yea.3812 36065479

[B39] Liu Z Wang MM Wang GS Li AH Mu W Wang QM (2021) *Hanseniasporaterricola* sp. nov., an ascomycetous yeast isolated from tibet. International Journal of Systematic and Evolutionary Microbiology 71: 004741. 10.1099/ijsem.0.004741 33720007

[B40] Liu S Guo QC An ZR Hui FL (2023) *Danielozymapruni* sp. nov., an asexual yeast species isolated from insect frass. International Journal of Systematic and Evolutionary Microbiology 73: 1–5. 10.1099/ijsem.0.006124 37991229

[B41] Liu M Jiang YL Zhang YX Wang QM (2024a) *Nakazawaeatricholomae* f.a., sp. nov., a Novel Ascomycetous Yeast Species Isolated from Two Mushroom Species in China. Current Microbiology 81: 1–5. 10.1007/s00284-023-03600-w 38281277

[B42] Liu M Ding RX Zhang YX Li HZ Wang QM (2024b) *Wickerhamomycescorioli* f.a., sp. nov., a novel yeast species discovered in two mushroom species. International Journal of Systematic and Evolutionary Microbiology 74: 1–8. 10.1099/ijsem.0.006333 38591772

[B43] Liu S Cai DY Hui FL (2024c) *Cyberlindneraqingyuanensis* f.a., sp. nov., a yeast species isolated from rotting wood. International Journal of Systematic and Evolutionary Microbiology 74: 2–6. 10.1099/ijsem.0.006507 39207228

[B44] Lu YF Chai CY Hui FL (2024) Two new phyllospheric species of *Colacogloea* (Colacogloeaceae, Pucciniomycotina) identified in China. MycoKeys 101: 81–94. 10.3897/mycokeys.101.114872 38250087 PMC10799300

[B45] Marcellino N Beuvier E Grappin R Guéguen M Benson DR (2001) Diversity of *Geotrichumcandidum* strains isolated from traditional cheesemaking fabrications in France. Applied and Environmental Microbiology 67: 4752–4759. 10.1128/AEM.67.10.4752-4759.2001 11571181 PMC93228

[B46] McNeill J Barrie FR Buck WR (2012) International Code of Nomenclature for algae, fungi and plants (Melbourne Code) adopted by the Eighteenth International Botanical Congress Melbourne, Australia, July 2011. Koeltz Botanical Books, Germany.

[B47] Nielsen DS Jakobsen M Jespersen L (2010) *Candidahalmiae* sp. nov., *Geotrichumghanense* sp. nov. and *Candidaawuaii* sp. nov., isolated from Ghanaian cocoa fermentations. International Journal of Systematic and Evolutionary Microbiology 60: 1460–1465. 10.1099/ijs.0.016006-0 19671722

[B48] Noster J Koeppel MB Desnos-Olivier M Aigner M Bader O Dichtl K Gottig S Haas A Kurzai O Pranada AB Stelzer Y Walther G Hamprecht A (2022) Bloodstream infections caused by *Magnusiomycescapitatus* and *Magnusiomycesclavatus*: epidemiological, clinical, and microbiological features of two emerging yeast species. Antimicrobial Agents and Chemotherapy 66: e01834-21. 10.1128/aac.01834-21 PMC8846490 34930027

[B49] O’Donnell K (1993) *Fusarium* and its near relatives. In: Reynolds DR Taylor JW (Eds) The fungal holomorph: mitotic, meiotic and pleomorphic speciation in fungal systematics.CAB International, 225–233.

[B50] Özkaya-Parlakay A Cengiz AB Karadaǧ-Öncel E Kuşkonmaz B Saribaş Z Kara A Oǧuz B (2012) *Geotrichumcapitatum* septicemia in a hematological malignancy patient with positive galactomannan antigen: Case report and review of the literature. Turkish Journal of Pediatrics 54: 674–678. 23692800

[B51] Pan F Qiu S Lv Y Li D (2023) Exploring the controllability of the Baijiu fermentation process with microbiota orientation. Food Research International 173: 113249. 10.1016/j.foodres.2023.113249 37803561

[B52] Posada D Crandall KA (1998) Modeltest: testing the model ofDNA substitution. Bioinformatics 9: 817–818. 10.1093/bioinformatics/14.9.817 9918953

[B53] Qiao YZ Chen X Hui FL (2023) *Barnettozymamenglunensis* f.a., sp. nov., a novel yeast species isolated from rotting wood. International Journal of Systematic and Evolutionary Microbiology 73: 1–5. 10.1099/ijsem.0.005711 36790429

[B54] Qiao YZ Liu S Niu QH Hui FL (2024) Three new *Dioszegia* species (Bulleribasidiaceae, Tremellales) discovered in the phylloplane in China. MycoKeys 101: 313–328. 10.3897/mycokeys.101.117174 38343719 PMC10858432

[B55] Rathnayaka AR Tennakoon DS Jones GE Wanasinghe DN Bhat DJ Priyashantha AH Stephenson SL Tibpromma S Karunarathna SC (2024) Significance of precise documentation of hosts and geospatial data of fungal collections, with an emphasis on plant-associated fungi. New Zealand Journal of Botany 1–28. 10.1080/0028825X.2024.2381734

[B56] Ronquist F Teslenko M Van Der Mark P Ayres DL Darling A Höhna S Larget B Liu L Suchard MA Huelsenbeck JP (2012) Mrbayes 3.2: Efficient bayesian phylogenetic inference and model choice across a large model space. Systematic Biology 61: 539–542. 10.1093/sysbio/sys029 22357727 PMC3329765

[B57] Shah A Mauger T (2017) *Magnusiomycescapitatus*: a new and emerging pathogen linked to keratomycosis. Digital journal of ophthalmology 23: 75–77. 10.5693/djo.02.2017.04.001 29162991 PMC5683434

[B58] Shi CF Zhang KH Chai CY Yan ZL Hui FL (2021) Diversity of the genus *Sugiyamaella* and description of two new species from rotting wood in China. MycoKeys 77: 27–39. 10.3897/mycokeys.77.60077 33519267 PMC7815693

[B59] Stamatakis A (2006) RAxML-VI-HPC: Maximum likelihood-based phylogenetic analyses with thousands of taxa and mixed models. Bioinformatics 22: 2688–2690. 10.1093/bioinformatics/btl446 16928733

[B60] Sulo P Laurenčík M Poláková S Minárik G Sláviková E (2009) *Geotrichumbryndzae* sp. nov., a novel asexual arthroconidial yeast species related to the genus *Galactomyces*. International Journal of Systematic and Evolutionary Microbiology 59: 2370–2374. 10.1099/ijs.0.008938-0 19605724

[B61] Swofford DL (2003) PAUP*: Phylogenetic analyses using parsi- mony. Version 4.0 beta. Sinauer Associates, Sunderland, MA.

[B62] Takei K Mitoma C Hashimoto-Hachiya A Takahara M Tsuji G Nakahara T Furue M (2015) *Galactomyces* fermentation filtrate prevents T helper 2-mediated reduction of filaggrin in an aryl hydrocarbon receptor-dependent manner. Clinical and Experimental Dermatology 40: 786–793. 10.1111/ced.12635 25786502

[B63] Tan YP Shivas RG (2023) Index of Australian Fungi no. 8 Nomenclatural novelties: Y.P. Tan & R.G. Shivas, 1–14. 10.5281/zenodo.8042624

[B64] Tan YP Shivas RG (2024) Index of Australian Fungi no. 41 Nomenclatural novelties: Yu Pei Tan & Roger G. Shivas, 1–22. 10.5281/zenodo.12816268

[B65] Tanuskova D Horakova J Svec P Bodova I Lengerova M Bezdicek M Poczova M Koppl J Kolenova A (2017) First case of invasive *Magnusiomycescapitatus* infection in Slovakia. Medical Mycology Case Reports 16: 12–15. 10.1016/j.mmcr.2017.03.004 28409093 PMC5379865

[B66] The Biodiversity Committee of Chinese Academy of Sciences (2024) Catalogue of Life China: 2024 Annual Checklist. Beijing, China.

[B67] Tshisevhe V Mitton B Skosana L (2021) Invasive *Geotrichumklebahnii* fungal infection: A case report. Access Microbiology 3: 1–4. 10.1099/acmi.0.000287 PMC8742591 35018329

[B68] Ueda-Nishimura K Mikata K (2000) Two distinct 18S rRNA secondary structures in *Dipodascus* (Hemiascomycetes). Microbiology 146: 1045–1051. 10.1099/00221287-146-5-1045 10832631

[B69] Vadkertiová R Molnárová J Vránová D Sláviková E (2012) Yeasts and yeast-like organisms associated with fruits and blossoms of different fruit trees. Canadian Journal of Microbiology 58: 1344–1352. 10.1139/cjm-2012-0468 23210991

[B70] Wang S Zhang H Qi T Deng L Yi L Zeng K (2022) Influence of arginine on the biocontrol efficiency of *Metschnikowiacitriensis* against *Geotrichumcitri-aurantii* causing sour rot of postharvest citrus fruit. Food Microbiology 101: 103888. 10.1016/j.fm.2021.103888 34579848

[B71] Wei XY Zhu HY Song L Zhang RP Li AH Niu QH Liu XZ Bai FY (2022) Yeast diversity in the Qaidam Basin Desert in China with the description of five new yeast species. Journal of Fungi 8: 858. 10.3390/jof8080858 PMC9409814 36012846

[B72] Wei YH Zhu HY Guo LC Luo LJ Lee CF Hui FL Han Y Zhen P Hu S Han PJ Bai FY (2024a) *Saturnisporasinensis* sp. nov., a new ascomycetous yeast species from soil and rotten wood. International Journal of Systematic and Evolutionary Microbiology 74: 006280. 10.1099/ijsem.0.006280 38415711

[B73] Wei YH Zhu HY Wen Z Guo LC Bai M Wang DQ Huang W Jiang LL Kajadpai N Srisuk N Han PJ Bai FY (2024b) *Starmerellafangiana* f.a. sp. nov., a new ascomycetous yeast species from Daqu-making environment and other sources. International Journal of Systematic and Evolutionary Microbiology. 74: 006581. 10.1099/ijsem.0.006581 PMC11578291 39565723

[B74] White TJ Bruns T Lee S Taylor J (1990) Amplification and direct sequencing of fungal ribosomal RNA genes for phylogenetics. PCR Protocols: a Guide to Methods and Applications 38: 315–322. 10.1016/B978-0-12-372180-8.50042-1

[B75] Xi ZW Wang XX Hui FL (2024) *Naohideaakebiae* fa. sp. nov., an anamorphic basidiomycete yeast species. International Journal of Systematic and Evolutionary Microbiology: 1–6. 10.1099/ijsem.0.006567 39475715

[B76] Yu HT Shang YJ Zhu HY Han PJ Wang QM Santos ARO Barros KO Souza GFL Alvarenga FBM Abegg MA Rosa CA Bai FY (2023) *Yueomycessilvicola* sp. nov., a novel ascomycetous yeast species unable to utilize ammonium, glutamate, and glutamine as sole nitrogen sources. Yeast 40: 1–10. 10.1002/yea.3901 37818980

[B77] Zhu HY Wei XY Liu XZ Bai FY (2023a) *Cystofilobasidiumjosepauloni*s sp. nov., a novel basidiomycetous yeast species. International Journal of Systematic and Evolutionary Microbiology 73: 005865. 10.1099/ijsem.0.005865 37191980

[B78] Zhu HY Wei YH Guo LC Wei XY Li JN Zhang RP Liu XZ Bai FY (2023b) *Vishniacozymapseudocarnescens* sp. nov., a new anamorphic tremellomycetous species. International Journal of Systematic and Evolutionary Microbiology 73: 006076. 10.1099/ijsem.0.006076 37847534

[B79] Zhu HY Han DY Guo LC Li JN Wei XY Zhang RP Wang QM Shang YJ Luo LJ Wei YH Liu XZ Bai FY (2023c) Diversity and distribution of yeasts in intertidal zones of China. Frontiers in Marine Science 10: 1286511. 10.3389/fmars.2023.1286511

[B80] Zhu HY Guo LC Hu S Wei YH Hui FL Liu XZ Bai FY (2024a) *Pichiakurtzmaniana* f.a. sp. nov., with the transfer of eight *Candida* species to *Pichia*. International Journal of Systematic and Evolutionary Microbiology: 1–8. 10.1099/ijsem.0.006306 PMC10995725 38536076

[B81] Zhu HY Shang YJ Wei XY Groenewald M Robert V Zhang RP (2024b) Taxonomic revision of *Geotrichum* and *Magnusiomyces*, with the descriptions of five new *Geotrichum* species from China. Mycology, 1–24. 10.1080/21501203.2023.2285764 PMC11376286 39247897

