# Supplementary figures and images for: ﻿Two new arthroconidial yeast species from bark and pit mud in China
Source: MycoKeys. 2025 Jan 28;113:57–72. doi: 10.3897/mycokeys.113.141799 (PMC11795185; doi:10.3897/mycokeys.113.141799)

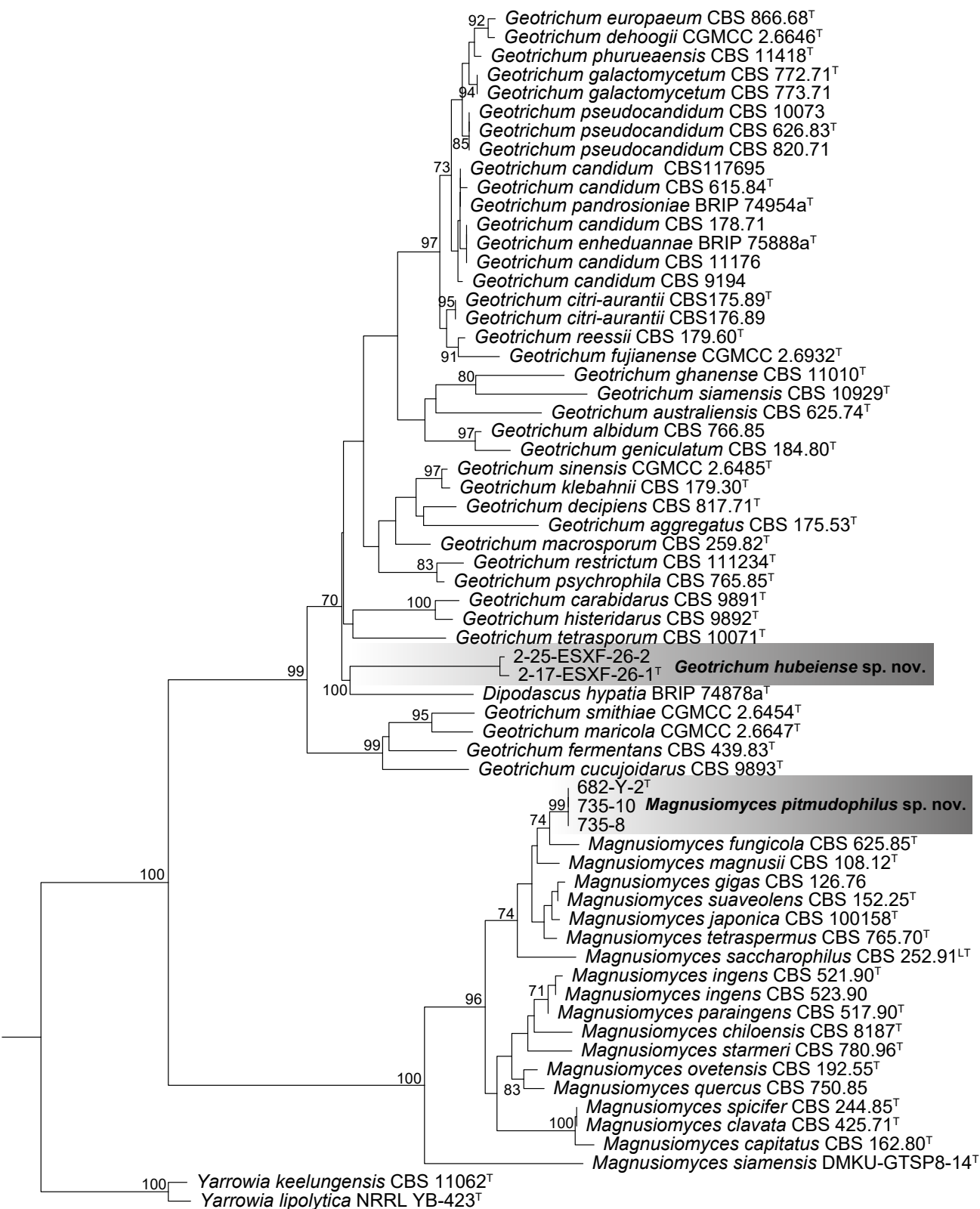

Supplement: Supplementary material 1 — Phylogeny of the described arthroconidial yeast species based on neighbor-joining (NJ) analysis of the D1/D2 sequences [file mycokeys-113-057-s001.pdf]

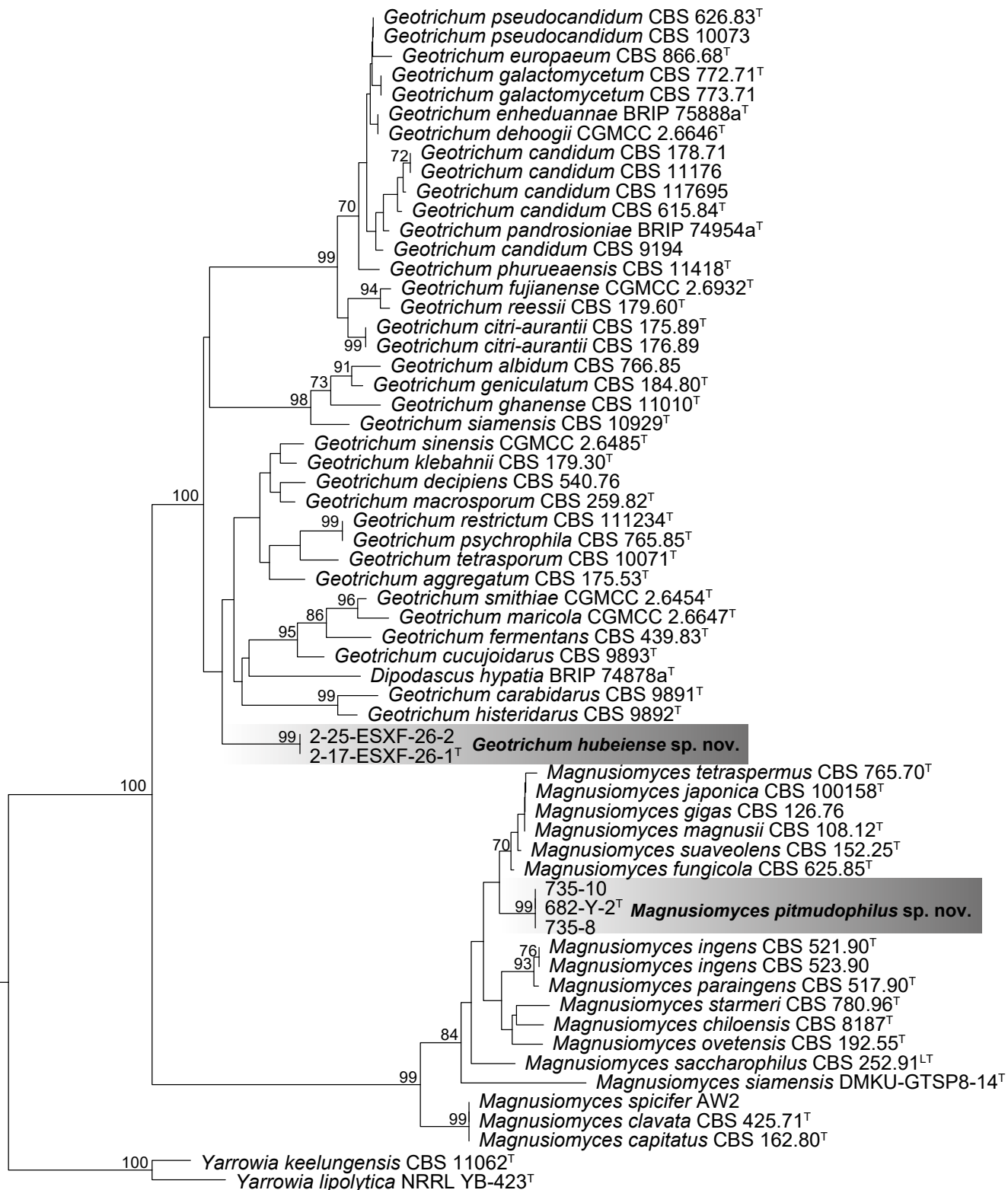

Supplement: Supplementary material 2 — Phylogeny of the arthroconidial yeast species based on neighbor-joining (NJ) analysis of the ITS sequences [file mycokeys-113-057-s002.pdf]
